# Follistatin-like 1 protects against hypoxia-induced pulmonary hypertension in mice

**DOI:** 10.1038/srep45820

**Published:** 2017-03-31

**Authors:** Wei Zhang, Wang Wang, Jie Liu, Jinna Li, Juan Wang, Yunxia Zhang, Zhifei Zhang, Yafei Liu, Yankun Jin, Jifeng Li, Jie Cao, Chen Wang, Wen Ning, Jun Wang

**Affiliations:** 1 Department of Physiology and Pathophysiology, Capital Medical University, Beijing 100069, P.R. China; 2 Beijing Key Laboratory of Respiratory and Pulmonary Circulation Disorders, Capital Medical University, Beijing 100069, P.R. China; 3 Respiratory Department, Tianjin Medical University General Hospital, Tianjin 300052, P.R. China; 4 Department of Respiratory and Critical Care Medicine, Beijing Chao-Yang Hospital, Capital Medical University, Beijing 100020, P.R. China; 5 Department of Respiratory and Critical Care Medicine, China-Japan Friendship Hospital, Beijing 100029, P.R. China; 6 State Key Laboratory of Medicinal Chemical Biology, College of Life Sciences, Nankai University, Tianjin 300071, P.R. China

## Abstract

Pulmonary hypertension (PH) remains a life-limiting disease characterized by pulmonary vascular remodelling due to aberrant proliferation and migration of pulmonary artery smooth muscle cells (PASMCs), thus leading to raised pulmonary arterial pressure and right ventricular hypertrophy. Secreted glycoprotein follistatin-like 1 (FSTL1) has been reported to ameliorate tissue remodelling in cardiovascular injuries. However, the role of FSTL1 in deranged pulmonary arteries remains elusive. We found that there were higher serum levels of FSTL1 in patients with PH related to chronic obstructive pulmonary diseases (COPD) and in mice model of hypoxia-induced PH (HPH). Haploinsufficiency of *Fstl1* in mice contributed to an exacerbated HPH, as demonstrated by increased right ventricular systolic pressure, pulmonary arterial muscularization and right ventricular hypertrophy index. Conversely, FSTL1 administration attenuated HPH. In cultured human PASMCs, hypoxia-promoted cellular viability, DNA synthesis and migration were suppressed by exogenous FSTL1 but enhanced by small interfering RNA targeting *FSTL1*. Additionally, FSTL1 inhibited the proliferation and migration of PASMCs via extracellular regulated kinase (ERK) signal pathway. All these findings indicate that FSTL1 imposed a protective modulation on pulmonary vascular remodelling, thereby suggesting its role in the regulation of HPH.

Pulmonary hypertension (PH), manifested by a sustained elevation of pulmonary arterial pressure (PAP) and right ventricular hypertrophy (RVH), is a devastating clinical disorder with an age-standardized mortality of 4.5–12.3 per 100,000 in USA^1^. The exact mechanism remains largely unknown and patients always die of right heart failure in spite of advances in pharmacological therapies over past years^2^,^3^. Pulmonary arterial remodelling, which comprises excessive proliferation of pulmonary artery smooth muscle cells (PASMCs) in the medial layer, is one of the most prominent features of PH^4^,^5^,^6^. Multiple risk factors are associated with PH. It is well accepted that chronic hypoxia stimulates vascular structural changes and lumen narrowing, leading to the alteration of pulmonary vascular responsiveness and contributing to the development of PH^7^. This occurs clinically in more than 30% of patients suffering from chronic obstructive pulmonary diseases (COPD), with a sharply rising death rate subsequently^8^,^9^. Actually, hypoxic rodent is one of the most commonly used models to dissect the molecular mechanisms and to identify potential therapeutic targets for PH^10^. Therefore, hypoxia exerts a pivotal role in PH pathogenesis. Unfortunately, no specific therapy is currently available to effectively reduce hypoxia-induced PH (HPH). The exploration for novel mediators or modulators to attenuate hypoxia-induced cellular responses and thus lessen abnormal vascular remodelling may be helpful to delay the progression of HPH.

Follistatin-like 1 (FSTL1) is a secreted glycoprotein initially induced by transforming growth factor-β1 (TGF-β1) from a mouse MC3T3-E1 osteoblast cell line^11^. It is widespread in mammalian tissues and produced mainly by cells of mesenchymal origin (fibroblasts, osteocytes, adipocytes, cardiomyocytes chondrocytes and trophoblasts)^12^,^13^. Additionally, endothelial cells (ECs), smooth muscle cells (SMCs), macrophages and epithelial cells are also cellular origins of FSTL1^14^,^15^,^16^. Disco-interacting protein 2 homolog A (DIP2A) has been suggested as a potential cell-surface receptor for FSTL1 in systemic ECs and cardiac myocytes^17^, yet to our knowledge, there seems no definite FSTL1 receptor in lung, where it functions in the extracellular matrix and regulates on other ligands^18^. Based on its extracellular calcium-binding and follistatin-like domains, FSTL1 belongs to the secreted protein acidic and rich in cysteine (SPARC) family. This family has been appreciated to play a critical role in organogenesis and human disease pathogenesis^19^. However, functions and mechanisms of FSTL1 have not been fully understood. A growing body of literatures have identified the regulatory functions of FSTL1 in cellular survival^15^,^20^, proliferation^16^,^21^, migration^16^,^21^,^22^,^23^ and differentiation^22^,^23^,^24^,^25^ involved both in physiological and pathological processes. Recently, beneficial effects of FSTL1 in cardiovascular diseases have been reported. In systemic vascular damages, FSTL1 could preserve the viability of ECs and SMCs both *in vivo* and *in vitro*^15^,^16^,^21^,^26^. In pulmonary circulation, it is highly expressed in blood vessels of the developing lung^27^, but its role in stressful arteries, such as in HPH, is not elucidated.

In this study, we reported the increased circulating levels of FSTL1 in patients with PH associated with COPD, as well as in hypoxia-challenged mice. Haplodeletion of *Fstl1* in mice aggravated HPH, whereas administration of recombinant human FSTL1 protein led to amelioration *in vivo*. We found that FSTL1 inhibited the proliferation and migration of PASMCs via extracellular regulated kinase (ERK) signal pathway. Our finding suggested a protective role of FSTL1 in pulmonary vascular remodelling, pointing to its potential clinical value for patients with HPH.

## Results

### FSTL1 is upregulated in patients with PH related to COPD and mice exposed to hypoxia

**Figure 1:**
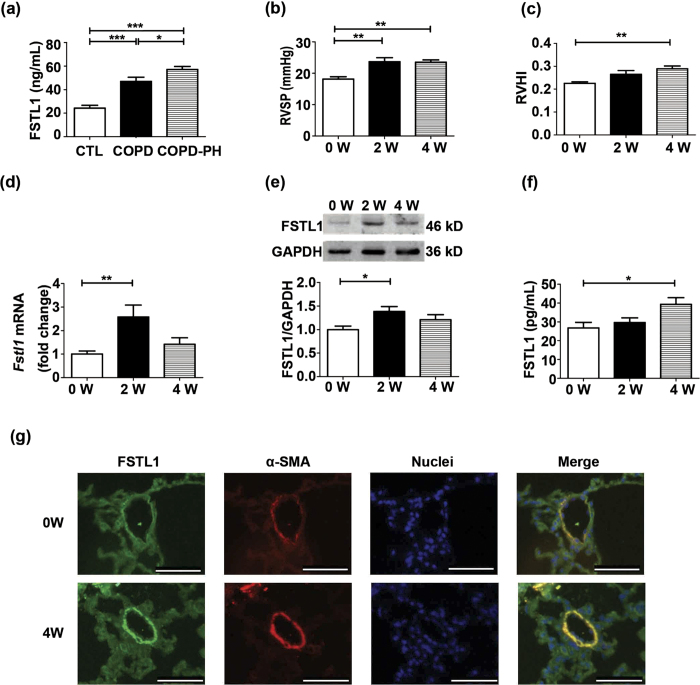
FSTL1 is upregulated in patients with PH related to COPD and mice exposed to hypoxia. (**a**) Serum concentration of FSTL1 protein by ELISA in patients with COPD only (n = 8), COPD combined with PH (n = 8) and healthy controls (CTL, n = 7). (**b**) Effect of chronic hypoxia on RVSP and RVHI (**c**) in C57BL/6 mice. n = 8. (**d**) QRT-PCR analysis of *Fstl1* mRNA in lung tissue of C57BL/6 mice under hypoxia as normalized by *Gapdh* mRNA. n = 10. (**e**) Representative cropped western blots and statistical analysis of FSTL1 protein in lung tissue of C57BL/6 mice under hypoxia as normalized by GAPDH. n = 10. (**f**) Serum concentration of FSTL1 protein by ELISA in C57BL/6 mice under hypoxia. n = 7–11. (**g**) Representative immunofluorescence images showing FSTL1 (green) and α-SMA (red) staining of pulmonary arterioles from lung sections in hypoxia-treated mice and untreated ones. Nuclei were stained with DAPI (blue). n = 4–5. Bar = 50 μm. Data are presented as mean ± SEM. *P < 0.05, **P < 0.01. COPD = chronic obstructive pulmonary diseases. PH = pulmonary hypertension. RVSP = right ventricular systolic pressure. RVHI = right ventricular hypertrophy index. W = week. ELISA = enzyme-linked immunosorbent assay. GAPDH = glyceraldehyde-3-phosphate dehydrogenase. α-SMA = α-smooth muscle actin. DAPI = 4′,6-diamidino-2-phenylindole.

We first determined whether FSTL1 production is aberrant in HPH patients. We analyzed serum FSTL1 concentrations from patients with COPD only or COPD combined with PH, and their healthy controls by enzyme-linked immunosorbent assay (ELISA). General characteristics of subjects were shown in Supplementary Table S1. As shown in Fig. 1a, COPD patients had a higher serum FSTL1 level as compared with healthy controls (P < 0.0001), which was significantly increased when complicated with PH (P = 0.0356). Subsequently, we setup a hypoxia mouse model of PH and measured the substantially elevated right ventricular systolic pressure (RVSP) and right ventricular hypertrophy index (RVHI) by week 2 and week 4, respectively, after hypoxia exposure (Fig. 1b and c, P < 0.01 for both RVSP and RVHI compared to untreated mice). The effects of hypoxia treatment on *Fstl1* mRNA expression were examined by quantitative real-time reverse transcription-polymerase chain reaction (qRT-PCR). As shown in Fig. 1d, hypoxia exposure increased *Fstl1* mRNA levels in lung tissues to 2.6 folds by week 2 (P < 0.01 compared to untreated mice) and to 1.4 folds by week 4 (P > 0.05 compared to untreated mice). Western blot analysis confirmed that the increase in *Fstl1* mRNA levels by hypoxia was accompanied with an increase to 1.4 folds in FSTL1 protein expression by week 2 (Fig. 1e, P < 0.05 compared to untreated mice). Serum collections from hypoxia-treated mice were also assayed for FSTL1 levels by ELISA. Figure 1f shows a remarkable elevation of 1.5 folds in circulating FSTL1 levels in mice after 4 weeks of hypoxia treatment (P < 0.05 compared to untreated mice). Consistently, immunofluorescent (IF) staining showed the higher level of FSTL1 protein in small remodelled pulmonary arteries (PAs) as compared to normal controls, which overlapped with α-smooth muscle actin (α-SMA), a specific marker for SMCs, suggesting that PASMCs could produce and secrete FSTL1 in adult mice (Fig. 1g). Above all, both human and mice data imply that FSTL1 is a HPH-related gene and may affect the pathogenesis of HPH.

### *Fstl1* ^+/−^ mice have an aggravated HPH phenotype after hypoxia treatment

**Figure 2:**
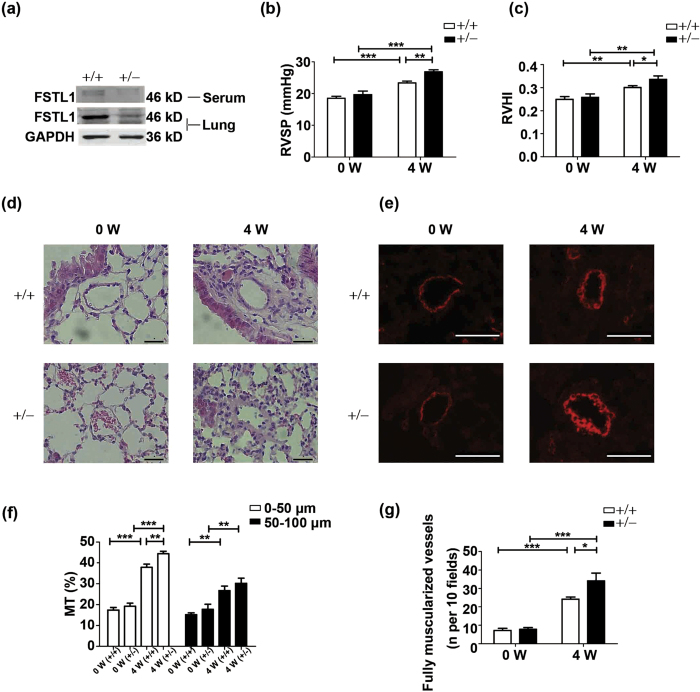
*Fstl1*^+/−^ mice have an aggravated HPH phenotype after hypoxia treatment. (**a**) Representative cropped western blots of FSTL1 protein in *Fstl1*^+/−^ and WT mice as normalized by GAPDH. n = 6. (**b**) RVSP in *Fstl1*^+/−^ and WT mice under hypoxia. n = 7–12. (**c**) RVHI in *Fstl1*^+/−^ and WT mice under hypoxia. n = 8–10. (**d**) Representative images showing hematoxylin and eosin staining of pulmonary arterioles from lung sections in *Fstl1*^+/−^ and WT mice under hypoxia. n = 4–5. Bar = 20 μm. (**e**) Representative immunofluorescence images showing α-SMA staining (red) of pulmonary arterioles from lung sections in *Fstl1*^+/−^ and WT mice under hypoxia. n = 4–5. Bar = 50 μm. (**f**) MT% of pulmonary arteries grouped by 0–50 μm and 50–100 μm in outer diameter from lung sections in *Fstl1*^+/−^ and WT mice under hypoxia. n = 5. (**g**) Numbers of completely muscularized arterioles (0–50 μm in outer diameter) per 10 fields from lung sections in *Fstl1*^+/−^ and WT mice under hypoxia. n = 5. Data are presented as mean ± SEM. *P < 0.05, **P < 0.01, ***P < 0.001. HPH = hypoxia-induced PH. WT = wide type. RVSP = right ventricular systolic pressure. RVHI = right ventricular hypertrophy index. MT = media thickness. W = week. GAPDH = glyceraldehyde-3-phosphate dehydrogenase. α-SMA = α-smooth muscle actin.

To investigate the biological significance of the inducible expression of FSTL1 during HPH, we examined the hypoxic response in *Fstl1*-deficient mice. Because homozygous *Fstl1*^*−/−*^ mice die of respiratory failure shortly after birth^18^, heterozygous *Fstl1*^+/–^ mice were used to study the hypoxic response. General characteristics of *Fstl1*^+/−^ and wide type (WT) mice were presented in Supplementary Table S2, with mean arterial pressure (MAP), as a surrogate for systemic pressure, not affected by FSTL1. *Fstl1*^+/–^ mice made significant less FSTL1 protein in lung tissue (~59% decrease) and displayed less circulating FSTL1 levels (~80% decrease), as compared to WT controls (Fig. 2a). As expected, both WT and *Fstl1*^+/−^ mice responded to hypoxia, as indicated with the elevations of both RVSP (Fig. 2b, P < 0.0001 and P = 0.0002 for WT and *Fstl1*^+/−^ mice, respectively) and RVHI (Fig. 2c, P = 0.0020 and P = 0.0025 for WT and *Fstl1*^+/−^ mice, respectively). But, *Fstl1*^+/–^ mice were more susceptible to hypoxia-induced lung injury and showed an increase of RVSP and RVHI than that of WT mice (Fig. 2b and c, P = 0.0013 for RVSP and P = 0.0493 for RVHI, respectively).

The extent of structural changes in pulmonary arterioles, characterized by de novo muscularization of precapillary pulmonary arteries, medial hypertrophy and intimal proliferation, is an important determinant for the severity of chronic HPH^7^,^28^. To examine whether the aggravated HPH phenotype in *Fstl1*^+/−^ mice is associated with the derangements of pulmonary arteries, we performed haematoxylin-eosin (HE) staining on lung sections. As shown in Fig. 2d, hypoxia-induced remodelling of pulmonary arterioles was much thicker in *Fstl1*^+/−^ mice. Furthermore, IF analysis of α-SMA showed the increased positive cells in *Fstl1*^+/−^ mice (Fig. 2e), indicating the proliferation of SMCs located in arterial media. Quantification of hypoxic lung sections by a blinded pathologist illustrated the increased thickness of media wall (MT%) in arteries of 0–100 μm diameters from both WT and *Fstl1*^+/−^ mice, respectively, after 4 weeks hypoxia treatment. Importantly, *Fstl1*^+/−^ mice displayed a more severe thickening media in arterioles smaller than 50 μm, as compared to WT mice (Fig. 2f, P = 0.0087). For larger arteries of 50–100 μm, hypoxia-induced MT% was higher in *Fstl1*^+/−^ mice than in WT ones, but the difference did not reach a statistical significance (Fig. 2f, P = 0.3231). This is in line with the fact that vascular remodelling is incited in smaller and more distant arterioles^4^. To further elucidate the effects of FSTL1 on de novo muscularization of arterioles, fully muscularized arterioles with external diameter <50 μm were detected and totaled per ten fields from each treatment group. The results exhibited a remarkable increase after 4 weeks of hypoxia, which was higher in *Fstl1*^+/−^ mice than WT ones (Fig. 2g, P = 0.0499). These *in vivo* data indicate that FSTL1 may be a critical homeostatic regulator in the pathogenesis of HPH and its deficiency could aggravate HPH.

### Administration of FSTL1 in mice leads to an attenuated HPH after hypoxia treatment

**Figure 3:**
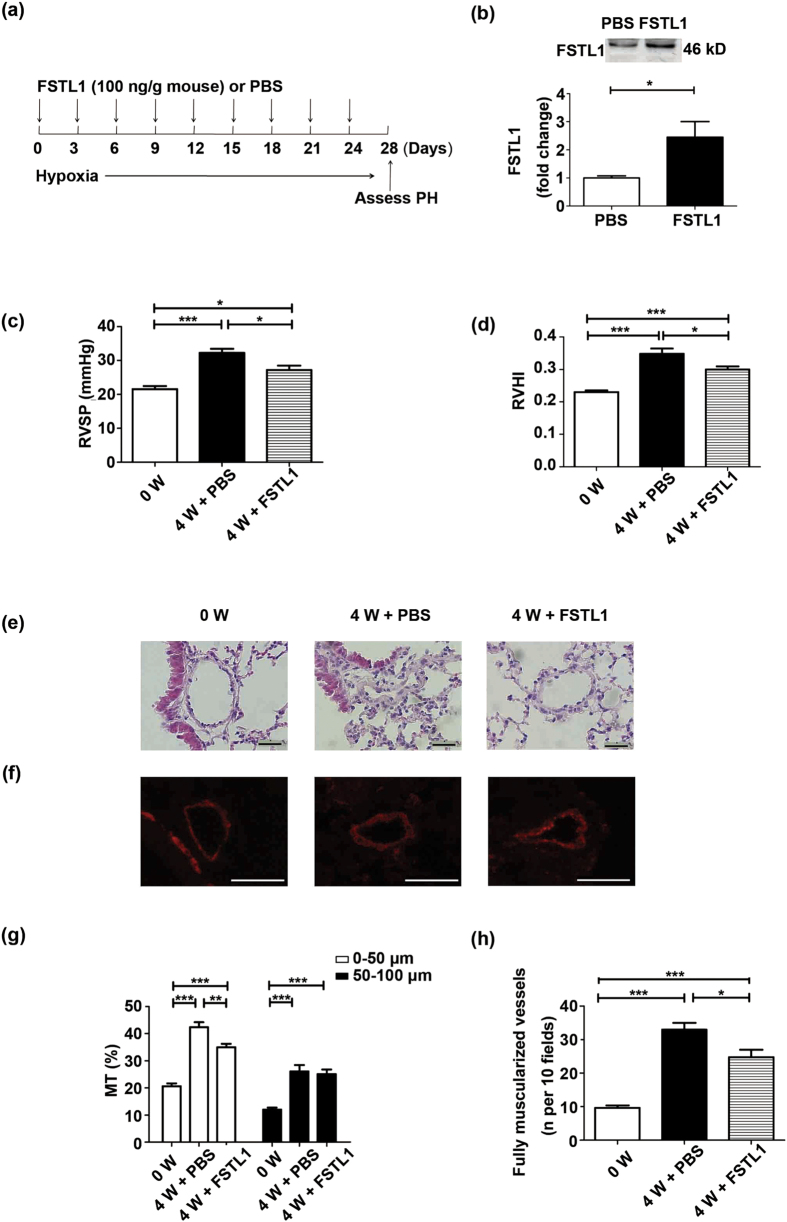
Administration of FSTL1 in mice leads to an attenuated HPH after hypoxia treatment. (**a**) FSTL1 treatment regimen in HPH model of mice. (**b**) Representative cropped western blots of serum FSTL1 protein in mice intravenously administrated with FSTL1 or PBS under hypoxia. n = 4. RVSP (**c**) and RVHI (**d**) in mice intravenously administrated with FSTL1 or PBS under hypoxia. n = 5. (**e**) Representative images showing hematoxylin and eosin staining of pulmonary arterioles from lung sections in mice intravenously administrated with FSTL1 or PBS under hypoxia. n = 4–5. Bar = 20 μm. (f) Representative immunofluorescence images showing α-SMA staining (red) of pulmonary arterioles from lung sections in mice intravenously administrated with FSTL1 or PBS under hypoxia. n = 4–5. Bar = 50 μm. (g) MT% of pulmonary arteries grouped by 0–50 μm and 50–100 μm in outer diameter from lung sections in mice intravenously administrated with FSTL1 or PBS under hypoxia. n = 5. (**h**) Numbers of completely muscularized arterioles (0–50 μm in outer diameter) per 10 fields from lung sections in mice intravenously administrated with FSTL1 or PBS under hypoxia. n = 5. Data are presented as mean ± SEM. *P < 0.05, **P < 0.01, ***P < 0.001. HPH = hypoxia-induced PH. PBS = phosphate buffer saline. RVSP = right ventricular systolic pressure. RVHI = right ventricular hypertrophy index. α-SMA = α-smooth muscle actin. MT = media thickness. W = week.

To verify our observation, recombinant human FSTL1 protein was administrated to C57BL/6 mice via tail-vein injection at the indicated time-points during hypoxia treatment (Fig. 3a). The dose we chose is according to an earlier observation that intravenous delivery of recombinant human FSTL1 100 ng/g (mouse) has led to a circulating concentration at 232 ng/mL^20^, similar to that effective to inhibit platelet derived growth factor (PDGF)-induced proliferative responses in cultured human aorta SMCs (HASMCs)^21^. The protocol for continual administration of FSTL1 protein is referred to an earlier study in which FSTL1-neutralizing antibody was given every 3 days to justify the interventional impact of FSTL1 on bleomycin-induced lung fibrosis in C57BL/6 mice^29^. General characteristics of mice were listed in Supplementary Table S3. As expected, we measured a 2.4-fold increase of serum concentration in mice treated with FSTL1 than phosphate buffer saline (PBS) (Fig. 3b, P = 0.0408). As shown in Fig. 3c and d, exogenous FSTL1 could attenuate HPH, as indicated by a reduction in RVSP and RVHI relative to PBS control (P = 0.0205 for RVSP and P = 0.0368 for RVHI, respectively).

In accordance with the attenuated RVSP level by administration of FSTL1, pulmonary morphometrics also displayed an ameliorated luminal narrowing and medial muscularization (Fig. 3e and f). Accordingly, a decrease in MT% was exhibited in FSTL1-treated arterioles of <50 μm (Fig. 3g, P = 0.0014) and the number of fully muscularized vessels also showed a drastic decline as compared to PBS controls (Fig. 3h, P = 0.0260). So both hemodynamic and morphological features are in coincidence with the hypoxic responses of heterozygous *Fstl1*^+/−^ mice, supporting the proposal that FSTL1 may operate beneficially in hypoxia-induced pulmonary vascular remodelling and thus delay the development of HPH.

### FSTL1 attenuates hypoxia-induced proliferation and migration of HPASMCs

**Figure 4:**
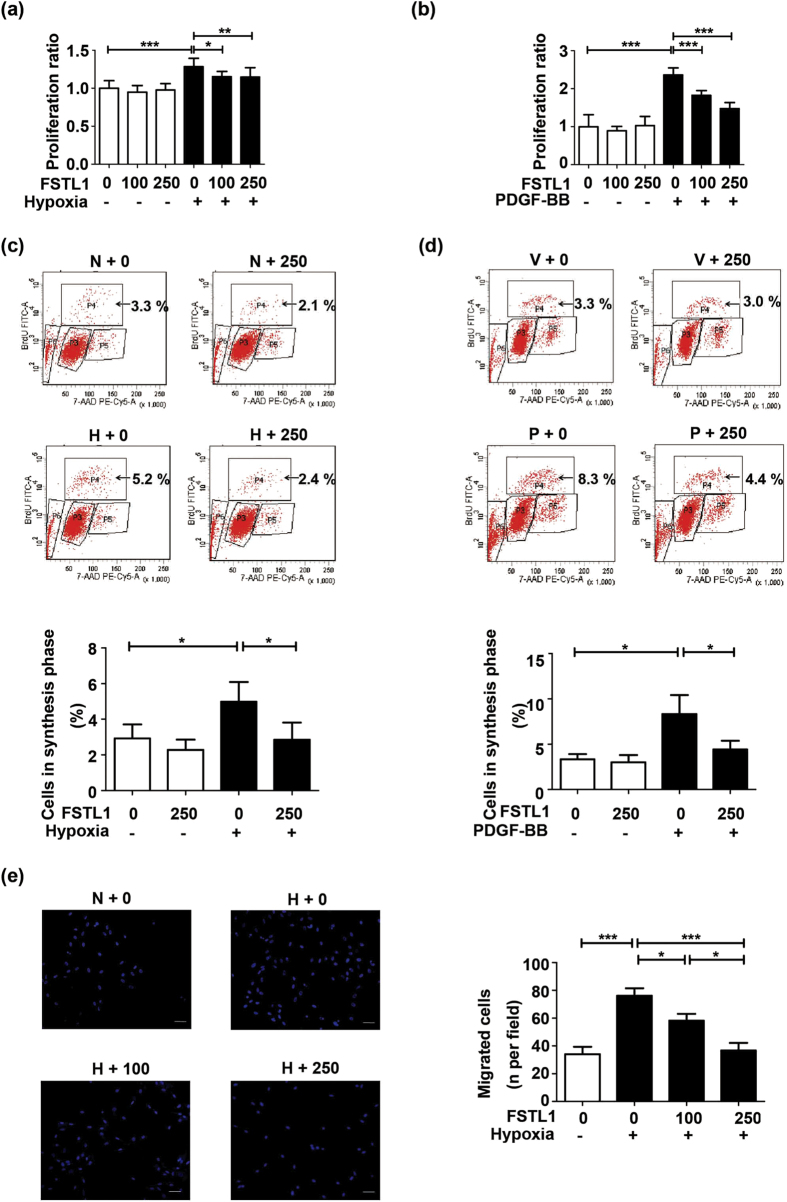
FSTL1 attenuates hypoxia-induced proliferation and migration of HPASMCs. (**a**) Effect of FSTL1 on cellular viability under hypoxia and PDGF-BB (**b**) in MTT assay. n = 3. (**c**) Effect of FSTL1 on DNA synthesis under hypoxia and PDGF-BB (**d**) in BrdU assay for flow cytometer analysis. Cells in synthesis phrase (S, P4) at a cell cycle was calculated as the percent of P4/(P3 + P4 + P5). n = 3. (**e**) Effect of FSTL1 on cellular migration in transwell chamber. Nuclei of trans-membrane cells were stained with DAPI (blue). n = 3. Bar = 50 μm. Data are presented as mean ± SEM. *P < 0.05, **P < 0.01, *** P < 0.001. HPASMCs = human pulmonary artery smooth muscle cells. P3 = G0/G1 phrase. P4 = S phase. P5 = G2 phrase. P6 = apoptosis phrase. MTT = 3-(4,5-dimethylthiazol-2-yl)-2,5-diphenyltetrazolium bromide. BrdU = 5-bromo-2-deoxyuridine. DAPI = 4’,6-diamidino-2-phenylindole. N = normoxia. H = hypoxia. V = vehicle. P = PDGF-BB. PDGF = platelet derived growth factor. FSTL1 = ng/mL.

To explore the efficacy of FSTL1 at a more mechanistic level, human PASMCs (HPASMCs) were pretreated with recombinant human FSTL1 or vehicle (PBS) followed by hypoxia challenge. FSTL1 concentrations of 100 ng/mL and 250 ng/mL were adopted as previously reported^20^,^21^,^30^. Cellular viability was determined by 3-(4,5-dimethylthiazol-2-yl)-2,5-diphenyltetrazolium bromide assay (MTT)^31^. As shown in Fig. 4a, 48 h of hypoxia led to an augmented HPASMCs proliferation (P < 0.0001 compared to baseline), which was obviously suppressed by FSTL1 (P < 0.05 for 100 ng/mL and P < 0.01 for 250 ng/mL). DNA synthesis in HPASMCs was analyzed by 5-bromo-2-deoxyuridine (BrdU) flow kit^32^. Figure 4c presents the flow cytometric analysis of cell subsets in different phases of cell cycle. Percentage of cells accumulated in synthesis (S, P4) phrase significantly increased under hypoxia (P = 0.0236 compared to baseline), but with a reduction when pretreated with FSTL1 (P = 0.0273). Chronic hypoxia also activates a variety of SMCs growth factors including PDGF^33^. The role of FSTL1 in PDGF-stimulated proliferation was also detected. HPASMCs were pretreated with recombinant human FSTL1 or vehicle (PBS) for 10 h followed by PDGF challenge (10 ng/mL) for 24 h. As can be seen in Fig. 4b, PDGF-induced cellular viability was obviously suppressed by FSTL1 (P < 0.001 for both 100 ng/mL and 250 ng/mL). Percentage of cells accumulated in synthesis (S, P4) phrase increased under PDGF (P = 0.0160 compared to baseline), but was significantly reduced when pretreated with FSTL1 (Fig. 4d, P = 0.0419). Our results implied the anti-proliferative capacity of FSTL1 in HPASMCs under hypoxic stimuli.

Transwell assay was performed to examine the effects of FSTL1 on cellular motility. Our findings revealed an obvious elevation in the number of migrated HPASMCs subjected to hypoxia (P < 0.0001 compared to baseline), which was suppressed by FSTL1 as compared to vehicle in a dose-dependent manner (Fig. 4e, P < 0.05 for 100 ng/mL and P < 0.0001 for 250 ng/mL, respectively). Taken together, FSTL1 could act as an anti-proliferation and anti-migration mediator in hypoxic HPASMCs.

### Small interfering RNA (siRNA)-mediated knock down of *FSTL1* promotes hypoxia-induced proliferation and migration of HPASMCs

**Figure 5:**
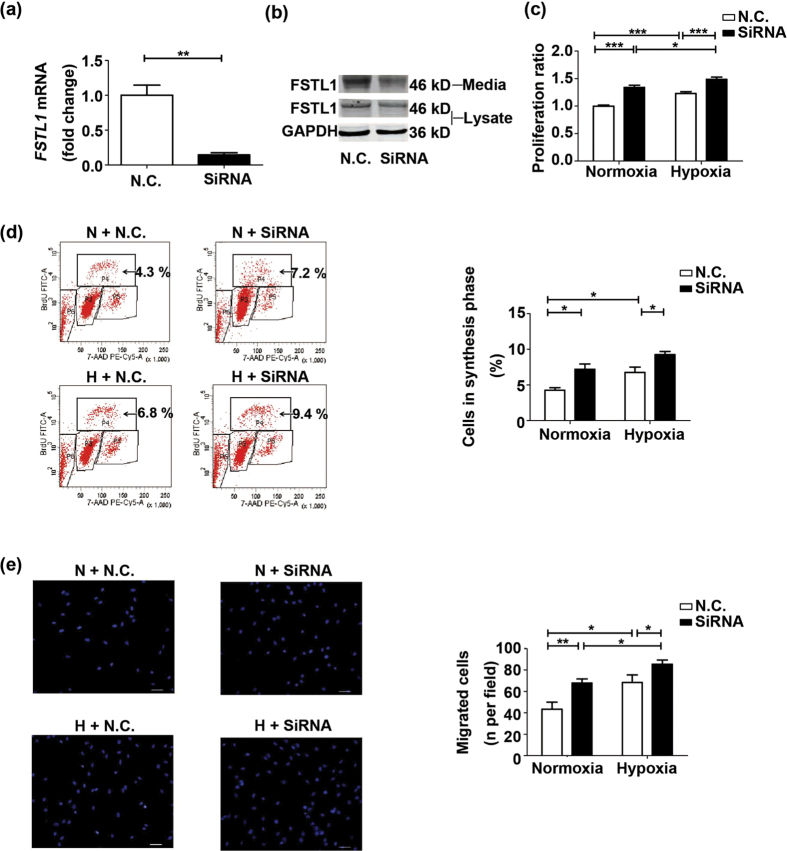
SiRNA-mediated knock down of *FSTL1* promotes hypoxia-induced proliferation and migration of HPASMCs. (**a**) QRT-PCR analysis of *FSTL1* mRNA in HPASMCs transfected with siRNA or N.C., as normalized by *GAPDH* mRNA. n = 4. (**b**) Representative cropped western blots of FSTL1 protein in HPASMCs transfected with siRNA or N.C. n = 3. (**c**) Effect of *FSTL1* siRNA transfection on cellular viability in MTT assay. n = 3. (**d**) Effect of *FSTL1* siRNA transfection on DNA synthesis in BrdU assay for flow cytometer analysis. Cells in synthesis phrase (S, P4) at a cell cycle was calculated as the percent of P4/(P3 + P4 + P5). n = 3. (**e**) Effect of *FSTL1* siRNA transfection on cellular migration in transwell chamber. Nuclei of trans-membrane cells were stained with DAPI (blue). n = 3. Bar = 50 μm. Data are presented as mean ± SEM. *P < 0.05, **P < 0.01, ***P < 0.001. SiRNA = small interfering RNA. N.C. = negative control. P3 = G0/G1 phrase. P4 = S phase. P5 = G2 phrase. P6 = apoptosis phrase. N = normoxia. H = hypoxia. GAPDH = glyceraldehyde-3-phosphate dehydrogenase. DAPI = 4′, 6-diamidino-2-phenylindole. MTT = 3-(4,5-dimethylthiazol-2-yl)-2,5-diphenyltetrazolium bromide. BrdU = 5-bromo-2-deoxyuridine.

To assess the significance of endogenous FSTL1, we knocked down *FSTL1* gene in the HPASMCs by siRNA transfection. The inhibition efficiency was determined by qRT-PCR and western blots, which proved a downregulation of FSTL1 at both mRNA (P = 0.0013) and protein levels, relative to negative control (N.C.) (Fig. 5a and b). On base of that, MTT assay showed the hypoxia-activated cellular growth was enhanced by *FSTL1* siRNA transfection as compared to N.C. in normoxic cells as well as cells subjected to hypoxia (Fig. 5c, P < 0.0001 for both). Similar results were obtained in BrdU incorporation experiment, with larger percentage of BrdU-incorporated cells by *FSTL1* knockdown than by N.C. controls under both normoxia and hypoxia (Fig. 5d, P = 0.0252 for normoxia and P = 0.0427 for hypoxia). In concordant, transwell assay discovered a larger number of hypoxia-treated cells allowed to migrate when treated with *FSTL1* siRNA than with N.C. (Fig. 5e, P = 0.0059 for normoxia and P = 0.0439 for hypoxia). These findings suggest endogenous FSTL1 as a homeostatic regulator in HPASMCs proliferation and migration, and thus provide supports as well as explanations for the beneficial effects of FSTL1 in HPH.

### ERK activity is implicated in the modulation of FSTL1 on HPH

**Figure 6:**
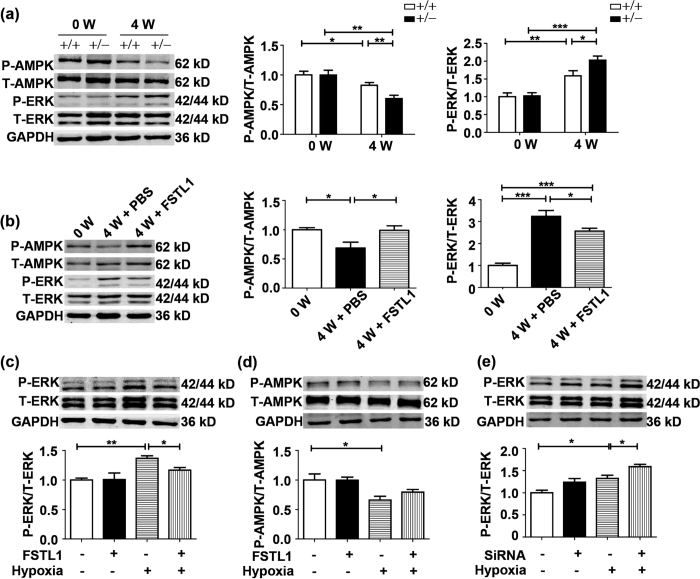
ERK activity is implicated in the modulation of FSTL1 on HPH. (**a**) Representative cropped western blots and statistical analysis of phosphorylations of AMPK (p-AMPK, n = 5) and ERK (p-ERK, n = 6) in lung tissue of *Fstl1*^+/−^ mice and WT controls under hypoxia. (**b**) Representative cropped western blots and statistical analysis of p-AMPK (n = 5) and p-ERK (n = 5) in lung tissue of mice treated with FSTL1 or PBS under hypoxia. Representative cropped western blots and statistical analysis of p-ERK (**c**) and p-AMPK (**d**) in HPASMCs exposed to hypoxia or normoxia for 24 h. n = 3. (**e**) Representative cropped western blots and statistical analysis of p-ERK in HPASMCs transfected with *FSTL1* siRNA exposed to hypoxia or normoxia for 24 h. n = 3. Data are presented as mean ± SEM. *P < 0.05, **P < 0.01, ***P < 0.001. HPH = hypoxia-induced PH. ERK = extracellular regulated kinase. AMPK = AMP-activated protein kinase. PBS = phosphate buffer saline. FSTL1 = 250 ng/mL. SiRNA = small interfering RNA. W = week.

To determine the molecular mechanism whereby FSTL1 alteration results in the phenotypes described earlier, we examined the phosphorylation levels (p) of Smad 1/5/8, mitogen-activated protein kinases (ERK, p38 kinase and Jun-N-terminal kinase) and AMP-activated protein kinase (AMPK), which are critical transducers in hypoxic pulmonary vascular remodelling. As can be drawn from Fig. 6a, immunoblotting detection of p-ERK showed a promoted signal by hypoxia compared to untreated mice (P = 0.0069 for WT and P < 0.0001 for *Fstl1*^+/−^ mice, respectively), which was further enhanced in *Fstl1*^+/−^ mice than in WT mice (P = 0.0345). Conversely, a reduction in p-AMPK occurred in hypoxic treated lungs (P = 0.0471 for WT and P = 0.0017 for *Fstl1*^+/−^ mice, respectively), with a further decrease in *Fstl1*^+/−^ mice as compared to WT mice (P = 0.0098). Accordingly, the hypoxia-stimulated phosphorylation level of ERK was attenuated by administration of FSTL1 in mice (Fig. 6b, P = 0.0428), accompanied with an exaggerated p-AMPK relative to PBS controls (P = 0.0443). However, assessment of p-Smad 1/5/8, p-p38 and p-JNK (Jun-N-terminal kinase) in *Fstl1*^+/−^ lungs revealed no significant differences compared with WT lungs in HPH mice (see Supplementary Fig. S1). Therefore, ERK and AMPK are among the potential signalling proteins participating in the modulation of FSTL1 against HPH.

**Figure 7:**
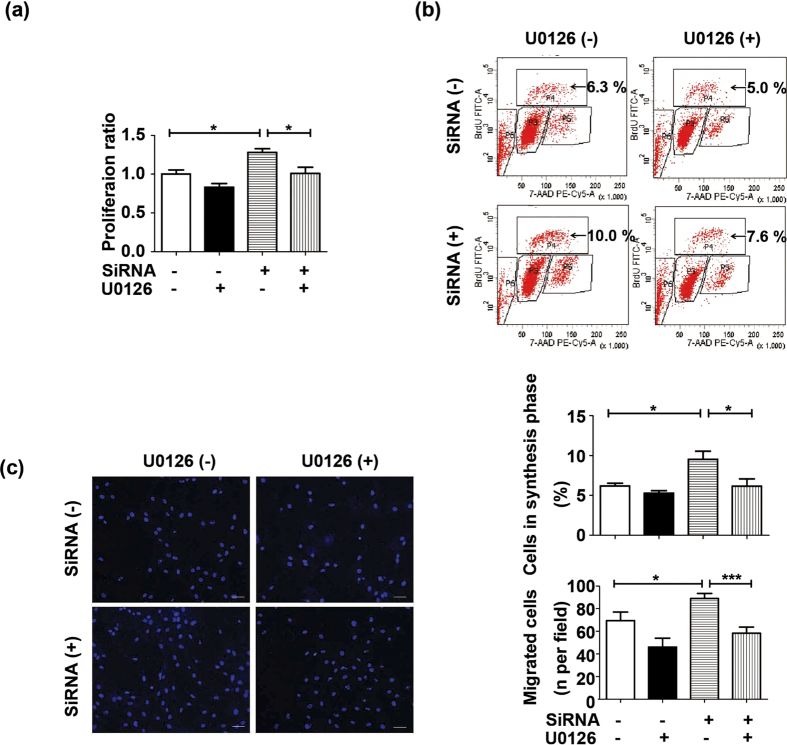
FSTL1 inhibited hypoxic HPASMCs proliferation and migration via ERK signalling. (**a**) Effect of ERK inactivation on *FSTL1* knockdown-induced cellular viability under hypoxia in MTT assay. n = 3. (**b**) Effect of ERK inactivation on *FSTL1* knockdown-induced DNA synthesis under hypoxia in BrdU assay for flow cytometer analysis. Cells in synthesis phrase (S, P4) at a cell cycle was calculated as the percent of P4/(P3 + P4 + P5). n = 3. (**c**) Effect of ERK inactivation on *FSTL1* knockdown-induced cellular migration under hypoxia in transwell chamber. Nuclei of trans-membrane cells were stained with DAPI (blue). n = 3. Bar = 50 μm. Data are presented as mean ± SEM. *P < 0.05, **P < 0.01, ***P < 0.001. ERK = extracellular regulated kinase. P3 = G0/G1 phrase. P4 = S phase. P5 = G2 phrase. P6 = apoptosis phrase. DAPI = 4′,6-diamidino-2-phenylindole. BrdU = 5-bromo-2-deoxyuridine. SiRNA = small interfering RNA. MTT = 3-(4,5-dimethylthiazol-2-yl)-2,5-diphenyltetrazolium bromide.

To testify whether FSTL1 directly affect ERK and AMPK signallings, FSTL1-pretreated HPASMCs were analyzed by western blots. FSTL1 (250 ng/mL) significantly suppressed the phosphorylated ERK under hypoxia (P = 0.0327 compared to vehicle), with total ERK levels (t-ERK) not affected (Fig. 6c). However, AMPK status in hypoxic HPASMCs was not influenced by FSTL1 (Fig. 6d, P = 0.1686 compared to vehicle). This implied that alteration of p-AMPK by FSTL1 may be in other types of vascular cells rather than in PASMCs. Accordingly, we found that siRNA knockdown targeting *FSTL1* could result in an activated p-ERK in hypoxic HPASMCs as compared to N.C. control (Fig. 6e, P = 0.0401). Furthermore, when pretreated with U0126, an ERK inhibitor, the elevated cellular viability and DNA synthesis in these cells were significantly prohibited (Fig. 7a and b, P = 0.0214 in MTT and P = 0.0382 in BrdU assay as compared to vehicle, respectively). Additionally, siRNA-treated HPASMCs in the presence of U0126 also exhibited lower chemotaxis (Fig. 7c, P < 0.001 compared to vehicle). These suggest a role of FSTL1 in HPASMCs by targeting ERK signalling.

## Discussion

Our research offered data at clinical, animal and cellular levels to support FSTL1, a mediator not previously associated with PH, as a novel homeostatic factor for HPH. Firstly, we showed that FSTL1 was upregulated in HPH. Secondly, the role of FSTL1 on HPH was suggested by the increased RVSP and pulmonary arterial remodelling in hypoxia-treated *Fstl1*^+/−^ mice relative to their WT littermates, with enhanced ERK phosphorylation and disrupted AMPK activity in lung tissue. We also demonstrated that systemic delivery of FSTL1 protein at least partly attenuated HPH in mice. Finally, we corroborated these data by finding that FSTL1 exerted a direct inhibitory impact on hypoxia-stimulated PASMCs proliferation and migration via ERK *in vitro*.

An earlier study has already demonstrated that FSTL1 protein expression could be markedly upregulated under hypoxia in primary human trophoblasts, just like NDRG1, a hypoxia-induced gene to alleviate hypoxic injury^13^. Most recently, emerging evidence has proposed FSTL1 as a clinically relevant secreted factor which could be highly regulated and impose a protective role in response to cardiovascular insults^34^,^35^. For patients with acute coronary syndrome (ACS)^24^,^36^ or heart failure^37^, FSTL1 was found to rise in circulation or in the explanted failing heart, respectively. Cardiac transcript level of *Fstl1* in mice has shown a substantial increase of 7 folds by transverse aortic constriction (TAC), which proved FSTL1 as an antihypertrophic “cardiokine” following pressure overload^30^. Additionally, serum FSTL1 was elevated to 3 folds by permanent left anterior descending coronary artery ligation (LAD), plus a 13-fold rise in transcript level of ischemic heart^35^. In this animal model, administration of exogenous FSTL1 has suppressed cardiac apoptosis and inflammation^20^. Moreover, FSTL1 has been identified as a novel “myokine” secreted by skeletal muscle to promote ischemic limb reperfusion or prohibit vascular neointimal formation^21^,^26^,^34^. All these are in supportive of our findings that FSTL1 was upregulated in patients and mice with deranged pulmonary arteries, with its significance being on the protection against PH. What’s more, a newly published study has revealed a remarkable positive association between plasma FSTL1 and derivatives of reactive oxidative metabolites (dROMs) concentrations in healthy male individuals^38^. DROMs can be used as a valuable marker for reactive oxygen species (ROS), the important mediators also linked with chronic hypoxia^39^. This strengthens our results by implying that FSTL1 could be induced by oxidative stress including chronic hypoxia. However, as we have shown in Fig. 3b, after 4 weeks of hypoxia challenge, FSTL1 level (in PBS controls) was even much less than that in mice intravenously administrated with exogenous FSTL1, which resulted in a partly improved HPH. So the insufficiency of hypoxia-induced FSTL1 may account for the reason why higher FSTL1 expression failed to protect against HPH. What’s more, it should be noted that after 4 weeks of hypoxia, both *Fstl1* mRNA and its protein level in lung tissue exhibited no significant difference as compared to baseline (Fig. 1d and e). Maybe for the sake of organ protection, FSTL1 was originally induced to confer resistance to damages caused by hypoxia. With the progression of HPH, the protection mechanisms would gradually come into a decompensation in lung and detrimental consequences occurred subsequently, leading to a resident reduction in *Fstl1* transcript and expression. Actually, the precise mechanism of serum FSTL1 elevation observed under hypoxic exposure remains unclear and should be explored further.

To the best of our knowledge, this study provides the first evidence that FSTL1 ameliorated hypoxia-induced vascular hypertrophy by suppression of PASMCs proliferation and migration. FSTL1 can be expressed in SMCs and ECs of embryonic vessels. Combined with the fact that it could prohibit the growth and metastatic potential of cancer cells, the mechanisms of FSTL1 action during vascular development may be to suppress cellular growth and invasion^40^,^41^,^42^. In pathological conditions, overexpression of FSTL1 prompted revascularization in murine ischemic limbs by protecting against endothelial cell apoptosis^26^. Also, FSTL1-mediated attenuation of neointimal thickening was demonstrated in rodent models of injurious carotid and femoral artery^16^,^21^. *In vitro* experiments proposed FSTL1 as an autocrine regulator which could be induced in human umbilical artery smooth muscle cells (HUASMCs) and negatively accumulate cells in G2 phase^16^. Combined with our results here, FSTL1 may play an important role in the control of aberrant structural remodelling in both systemic and pulmonary vascular diseases.

FSTL1 has been identified as an antagonist for bone morphogenetic protein 4 (BMP4) to negatively regulate Smad 1/5/8 signalling in lung morphogenesis and lung fibrosis^18^,^29^. Our study observed the discrepant results regarding p-Smads levels by FSTL1 between HPASMCs and human pulmonary artery endothelial cells (HPAECs), with FSTL1 limiting the activation of Smad 1/5/8 in HPAECs while exhibiting no effects in HPASMCs. This cell-specific function may explain why FSTL1 could negatively regulate BMP/Smads in PAECs, as has been demonstrated by Gosens’ lab^43^, but in the meantime, no changes had been detected in Smads levels by FSTL1 in total lung tissues of our HPH mice (see Supplementary Fig. S1). Previous studies have reported the impact of FSTL1 on ERK and AMPK activities in injurious myocyte and systemic vascular cells^20^,^21^,^35^. Our study demonstrated that haplodeletion of *Fstl1* in HPH mice led to a decreased p-AMPK but increased p-ERK in lung homogenate as compared to WT group, while systemic delivery of FSTL1 upregulated p-AMPK but downregulated p-ERK relative to PBS controls. ERK is a key enzyme that can be activated by hypoxia^44^. Blockade of ERK activation could inhibit HPASMCs proliferation and prevent hypoxia- and monocrotaline (MCT)-induced pulmonary vascular remodelling^45^,^46^. Conversely, AMPK is an energy sensor serving to switch off the rapid synthesis of protein, lipid and ribosomal RNA required for proliferating cells^47^. AMPK agonist metformin could improve hypoxia-induced or MCT-induced PH in rodents^48^. Also, endothelial-specific AMPK-knockout mice has developed an accelerated HPH^49^. All these findings offer clues about the involvement of ERK and AMPK signallings in FSTL1-mediated pulmonary arterial remodelling. Intriguingly, our *in vitro* experiment further indicated that FSTL1 affected HPASMCs via ERK but not AMPK signalling, which is different from HASMCs^21^. Discrepancy in responsiveness between HPASMCs and HASMCs could be one reason. Another plausible explanation is that endothelium constitutes the primary source of AMPK, which was crucial for the homeostasis of hypoxic pulmonary arteries partly by downregulation of adjacent PASMCs proliferation^49^. So it is likely that in HPH mice, FSTL1-orchestrated AMPK activity was predominantly detected in ECs but not in SMCs. Thus different roles of FSTL1 in multiple cell types should be in further careful consideration and additional studies are warranted.

The potential therapeutic effects of FSTL1 on HPH have also been tested by systemic administration of FSTL1 protein after 2 weeks of hypoxia (see Supplementary Fig. S2). Although the differences did not reach statistical significance, we have observed the trend of reduced HPH from FSTL1-treated mice as compared to PBS controls (P = 0.1282 for RVSP and P = 0.1176 for RVHI, respectively). It has been reported that pulmonary arterial remodelling occurs immediately after exposure to hypoxia, with cellular proliferation and medial hypertrophy peaking at 7 days and 10 days, respectively^50^. This may be the reason for our findings here and highlight the need for effective interference before establishment of active remodelling. Together with the earlier results that FSTL1 treatment starting from the onset of hypoxia has partly delayed the vascular derangement in mice, our findings have proposed the capacity of FSTL1 to improve HPH progression.

In conclusion, we attract renewed focus on the protective effects of FSTL1 on HPH. Although the significance of FSTL1 remains to be determined in other animal models, as well as in the clinical setting, our study has positively shown the rationale for further detecting the use of FSTL1 in PH.

## Methods

### Ethics declaration

This study obtained the written informed consents from all human subjects according to the Declaration of Helsinki, and the authorization from the Ethics Committees of Tianjin Medical University General Hospital and Beijing Chao-Yang Hospital of Capital Medical University. The human research protocol was approved by the Institutional Review Boards of both hospitals, and all experimental methods involving human subjects were completed in accordance with the relevant guidelines and regulations. All animal protocols were approved by the Institutional Animal Care and Use Committee of Capital Medical University, and complied with Regulations for the Management of Laboratory Animals announced by the Ministry of Science and Technology of People’s Republic of China.

### Subjects

Serum samples from patients with COPD only, COPD combined with PH, and their healthy controls matched in age, sex and smoking history were collected from Tianjin Medical University General Hospital and Beijing Chao-Yang Hospital in 2016. COPD was diagnosed according to the Global Initiative for Chronic Obstructive Lung Disease (GOLD) criteria. PH was determined by an echocardiography with the pulmonary arterial systolic pressure (PASP) no less than 38 mmHg^51^. Excluded were patients with valvular heart diseases, diabetes mellitus, bronchiectasis, pulmonary embolism or tuberculosis, tumor or inability to cooperate. Venous blood samples were allowed to clot for two hours at room temperature before centrifugation for 15 min at 3000 rotations per minute. Serum was stored in aliquot at −80 °C.

### Animal models

Male C57BL/6 mice (8–10 weeks) were purchased from Vital River Laboratory Animal Technology Company of Beijing in China. *Fstl1*^+/−^ mice and their WT littermates were a kindly gift from College of Life Sciences, Nankai University (Tianjin, China). All animals were specific pathogen free (SPF) with unrestricted standard mouse chew and water. After 3 days of acclimation, mice were randomly exposed to mixed air containing 10% oxygen in a normobaric chamber (BioSpherix, USA) for 0, 2 or 4 weeks. Cages were kept at 22–24 °C and opened for cleaning every 3 days for half an hour.

For systemic delivery of FSTL1 protein, mice were randomly subjected to tail-vein injection with recombinant human FSTL1 protein (100 ng/g mouse^20^, R & D Systems, USA) or with equal dosage of vehicle (PBS), immediately before the beginning of hypoxia or after 2 weeks of hypoxia, and repeated every 3 days until the end.

### Measurements of hemodynamics in HPH mice

After anesthetized with 2% pentobarbital (50 mg/kg, i.p.), mice were placed on a pad with chests shaved and disinfected. Pressure waveforms were detected by a closed-chest puncture into right ventricle (RV) and transduced to the PowerLab system (ADInstruments, Australia)^52^ for analysis of RVSP as a surrogate for mean PAP. Systemic blood pressure and heart rate (HR) were assessed by tail cuff method (Softron, Japan). Blood sample was then withdrawn by cardiac apex puncture for hematocrit analysis (Radiometer, Denmark). Serum was stored in aliquot at −80 °C.

Mice were euthanized by blood drain, with hearts and lungs flushed with saline and then dissected. The free wall of RV was excised from septum. Ratio of RV weight to left ventricle plus septum (LV + S) weight was calculated as RVHI.

### Analysis of lung morphometrics in HPH mice

Serial sections in thickness of 4 μm were cut through the paraffin-embedded left lung lobe and stained with HE (Beijing Dingguo Changsheng Biotechnology, China). Anti-α-SMA (1:200, Sigma-Aldrich, USA) and FSTL1 (1:200, Santa Cruz, USA) were visualized by Alexa Fluor 594-labelled goat anti-mouse IgG and Fluorescein-conjugated rabbit anti-goat IgG (ZsBio, China), respectively, with nuclei mounted by 4′,6-diamidino-2-phenylindole (DAPI, ZsBio, China). Blood vessels were screened with a microscope digital camera (Nikon, Japan) and analyzed by NIS-Elements system (Nikon, Japan). Vascular remodelling was evaluated by MT% and numbers of completely muscularized arterioles^52^,^53^. Briefly, MT% was expressed as a percentage of ((external diameter - internal diameter)/external diameter). Arterioles exhibiting more than 75% of circumference positive for α-SMA were identified as completely muscularized arteries and their numbers were totaled in every 10 high-power (×400) fields. Transversely cut arterioles were included for measurement, with the exclusion of obliquely cut ones and pulmonary veins.

### Cell culture

HPASMCs (4–8 passages, ScienCell Research Laboratories, USA) were cultured in complete smooth muscle cell medium (SMCM)^54^. Cells were placed in a CO_2_ incubator (5%, Thermo, USA) at 37 °C until reaching a confluence of 80%-90%. After starvation by fetal bovine serum (FBS)-free SMCM to arrest growth for 24 h, cells were pretreated with FSTL1 (100 ng/mL and 250 ng/mL) in 2% FBS-SMCM for 10 h, followed by stimulation under hypoxia (3% oxygen) or sometimes under PDGF-BB (10 ng/mL). For some experiments, U0126 (15 μM, Cell Signalling Technology, USA) was added 2 h before FSTL1 treatment.

### Cell proliferation

HPASMCs were seeded at 5 × 10^3^ cells per well in a 96-well plate and treated under different conditions. Numbers of cells were counted in MTT assay (Amresco, USA) as described before^54^. For analysis of DNA synthesis, a sample of 1 × 10^6^ cells were incubated with BrdU (10 μM) at 37 °C for 1 h. Collected cells were fixed and permeabilized, followed by exposure to DNase (300 μg per sample) at 37 °C for 1 h. The incorporated BrdU was stained with specific anti-BrdU fluorescent antibody at room temperature for 20 min. 7-AAD (20 μL per sample) was added for staining of total DNA. All reagents were from BD Pharmingen, USA. Detected cells in S phrase (P4), G0/G1 phrase (P3), G2 phrase (P5) and apoptosis phrase (P6) were separated and analyzed by an LSRFortessa flow cytometer (Becton Dickinson, USA). S phrase cells at a cell cycle was calculated as the percent of P4/(P3 + P4 + P5).

### Human *FSTL1* siRNA preparation

HPASMCs were seeded in a 96-well or 6-well plate at 60% confluence, followed by starvation for 24 h. *FSTL1* siRNA (GenePharma, China) and Lipofectamine RNAiMAX were diluted in Opti-MEM medium (Invitrogen, USA) and then mixed gentlely for 5 min incubation at room temperature. Then the siRNA-reagent mixture was added to every well at the final siRNA amount of 1 pmol in a 96-well plate and 25 pmol in a 6-well plate, respectively. The medium was changed for 2% FBS-SMCM after 6 h. Transfection efficiency was verified by qRT-PCR and western blot. The siRNA sequences were as following:

*FSTL1* siRNA: sense: 5′-GAAACUGCCAUCAAUAUUATT-3′;

anti-sense: 5′-UAAUAUUGAUGGCAGUUUCTT-3′.

N.C. siRNA: sense: 5′-UUCUCCGAACGUGUCACGUTT-3′;

anti-sense: 5′-ACGUGACACGUUCGGAGAATT-3′.

### Cell migration

Serum-deprived cells were seeded at 1 × 10^5^ per transwell chamber (Millipore, USA) in a 24-well plate and allowed to migrate through the 8.0 μm membrane pore for 6 h under hypoxia. Then upper surface of membrane was washed with PBS twice and swabbed with a cotton bud to discard non-migrated cells. Migrated cells on the lower surface were fixed in 4% paraformaldehyde for 20 min. DAPI-labelled nuclei were quantified in high-power fields under a microscope digital camera (Nikon, Japan).

### Western blot

Equal amounts of protein extracted from lung tissue, HPASMCs or plasma (1 μL) were subjected to sodium dodecyl sulfate-polyacrylamide gel electrophoresis (SDS-PAGE) and then transferred to a nitrocellulose membrane (0.45 μm, Millipore, USA). After blocked by 5% non-fat milk for at least 1 h at room temperature, the membrane was incubated in primary antibodies of FSTL1 (1:500, Santa Cruz, USA), glyceraldehyde-3-phosphate dehydrogenase (GAPDH, 1:2000, Cell Signalling Technology, USA), p-ERK, t-ERK, p-AMPK, t-AMPK, p-Smad 1/5/8, t-Smad 1/5/8, p-p38, t-p38, p-JNK and t-JNK (1:200, Cell Signalling Technology, USA) overnight at 4 °C. Then the membrane was washed in PBST (0.1% Tween 20-PBS, Sigma-Aldrich, USA) 3 × 10 min followed by incubation in IRDye800-conjugated secondary antibody (1:10000, Odyssey LI-COR, USA) for 1 h. After washed again in PBST 3 × 10 min, the membrane was visualized by the LI-COR Odyssey imaging system. The protein signal was quantified by Image J and relative protein level was normalized to that of GAPDH as house-keeping protein or to their respective total expressions.

### qRT-PCR

Lung lobe was incubated in RNAlater (Ambion, USA) at 4 °C overnight and stored at −80 °C. Total RNA was extracted from tissue or cultured HPASMCs with the Trizol reagents (Sigma-Aldrich, USA). Reverse-transcription was performed with Superscript III First-strand Synthesis System (Invitrogen, USA) and quantitative real-time PCR with Brilliant SYBRgreen QPCR Master Mix by a Mx3000 P System (Stratagene, USA), as previously conducted in our laboratory^31^. The relative abundance of *Fstl1* mRNA in mice or *FSTL1* mRNA in HPASMCs was normalized to that of the constitutively expressed *Gapdh* or *GAPDH*, respectively, by a comparative cycle threshold method (2^−ΔΔCT^). The primer sequences were listed below:

*Fstl1*: sense: 5′-TCCAAGATCCAGGTTGATTATGATG-3′;

anti-sense: 5′-TCGCGGTTAGCTTGATAGCAGA-3′.

*Gapdh*: sense: 5′-CGGAGTCAACGGATTTGGTCGTAT-3′;

anti-sense: 5′-AGCCTTCTCCATGGTGGTGAAGAC-3′.

*FSTL1*: sense: 5′-TCTGTGCCAATGTGTTTTGTGG-3′;

anti-sense: 5′-TGAGGTAGGTCTTGCCATTACTG-3′.

*GAPDH*: 5′-GGGTGTGAACCATGAGAAGTATGA-3′;

anti-sense: 5′-TGCTAAGCAGTTGGTGGTGC-3′.

### ELISA assay

Serum FSTL1 concentrations were analyzed by ELISA kits (Uscnk, China) for human and mice as instructed by manual.

### Statistical analysis

All data are expressed as mean ± standard error of mean (SEM) and analyzed using GraphPad Prism 5.0 (GraphPad sofware, USA). One-way analysis of variance (ANOVA) followed by Tukey’s Multiple Comparison was carried out for analysis of differences among 3 or more groups. Comparison between two groups was conducted using two-tailed unpaired Student’s *t* test. Statistical significance was considered as P < 0.05.

## Additional Information

**How to cite this article**: Zhang, W. *et al*. Follistatin-like 1 protects against hypoxia-induced pulmonary hypertension in mice. *Sci. Rep.* **7**, 45820; doi: 10.1038/srep45820 (2017).

**Publisher's note:** Springer Nature remains neutral with regard to jurisdictional claims in published maps and institutional affiliations.

## Supplementary Material

Supplementary Tables and Figures
