# Supplementary material for: Follistatin-like 1 protects against hypoxia-induced pulmonary hypertension in mice
Source: Sci Rep. 2017 Mar 31;7:45820. doi: 10.1038/srep45820 (PMC5374469; doi:10.1038/srep45820)

Follistatin-like 1 protects against hypoxia-induced pulmonary hypertension in mice

Wei Zhang, Wang Wang, Jie Liu, Jinna Li, Juan Wang, Yunxia Zhang, Zhifei Zhang, Yafei Liu, Yankun Jin, Jifeng Li, Jie Cao, Chen Wang [*], Wen Ning [*] & Jun Wang [*]

Supplementary Information

	CTL (n = 7)	COPD only (n = 8)	COPD with PH (n = 8)
Age (year)	70 ± 5	77 ± 1	74 ± 3
Male/Female (n)	3/4	5/3	3/5
BMI (kg/m^2)	24.2 ± 1.2	23.4 ± 2.6	24.9 ± 1.1
Smoking pack-years	23.4 ± 2.4	26.8 ± 5.1	38.5 ± 7.9
FEV_1/FVC (%)		53.1 ± 2.7	50.4 ± 3.6
PASP (mmHg)		26.0 ± 1.3	46.8 ± 2.4***

Supplementary Table S1. General characteristics of human subjects. CTL = healthy controls. COPD = chronic obstructive pulmonary diseases. PH = pulmonary hypertension. BMI = body mass index. FEV_1 = forced expiratory volume in one second. FVC = forced vital capacity. PASP = pulmonary arterial systolic pressure. Data are presented as mean ± SEM. *** $P < 0.001$ compared to COPD only.

	0 W		4 W	
	+/+	+/−	+/+	+/−
BW (g)	25.34 ± 0.72	26.87 ± 1.00	24.67 ± 0.75	24.15 ± 0.68*
HCT (%)	41.00 ± 1.40	42.50 ± 1.45	49.10 ± 1.15**	50.83 ± 2.06**
MAP (mmHg)	74.83 ± 3.15	72.60 ± 4.08	69.83 ± 2.61	71.17 ± 1.99
HR (BPM)	450.8 ± 13.08	449.0 ± 13.18	468.3 ± 6.33	451.7 ± 15.22

Supplementary Table S2. General characteristics of *Fstl1*$^{+/-}$ and WT mice under hypoxia. W = week. BW = body weight. HCT = hematocrit. MAP = mean arterial pressure. HR = heart rate. BPM = beats per minute. Data are presented as mean ± SEM. n = 6-10. * P < 0.05, ** P < 0.01 compared to untreated mice.

	0 W	4 W + PBS	4 W + FSTL1
BW (g)	22.80 ± 0.67	21.91 ± 0.50	22.40 ± 0.54
HCT (%)	43.25 ± 1.31	51.25 ± 1.54**	48.67 ± 0.33*
MAP (mmHg)	72.86 ± 3.31	68.00 ± 2.28	72.60 ± 3.12
HR (BPM)	442.5 ± 11.61	453.9 ± 11.87	462.7 ± 9.19

Supplementary Table S3. General characteristics of mice administrated with FSTL1 or PBS under hypoxia. W = week. BW = body weight. HCT = hematocrit. MAP = mean arterial pressure. HR = heart rate. BPM = beats per minute. PBS = phosphate buffer saline. Data are presented as mean ± SEM. n = 6-7. * $P < 0.05$, ** $P < 0.01$ compared to untreated mice.

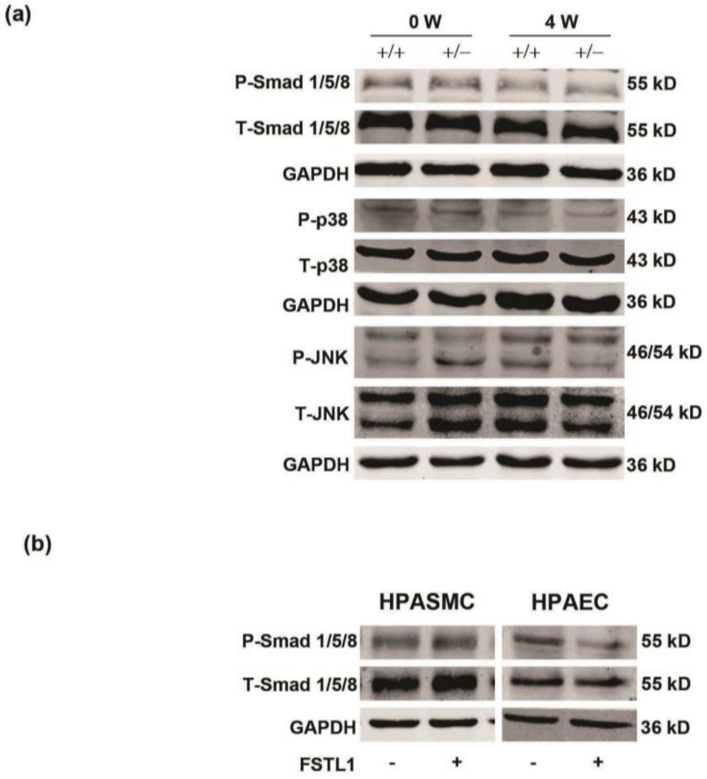

Supplementary Figure S1. Smad 1/5/8, p38 and JNK activities are not implicated in the modulation FSTL1 on HPH. (a) Representative western immunoblots for phosphorylations of Smad 1/5/8 (p-Smad 1/5/8), p38 (p-p38) and JNK (p-JNK) in lung tissue of *Fstl1*$^{+/-}$ mice and WT controls under hypoxia. n = 6. (b) Representative cropped western immunoblots for phosphorylations of Smad 1/5/8 (p-Smad 1/5/8) in hypoxic HPASMCs and HPAECs. HPH = hypoxia-induced pulmonary hypertension. WT = wide type. HPASMCs = human pulmonary artery smooth muscle cells. HPAECs = human pulmonary artery endothelial cells. JNK = Jun-N-terminal kinase. GAPDH = glyceraldehyde-3-phosphate dehydrogenase. W = week.

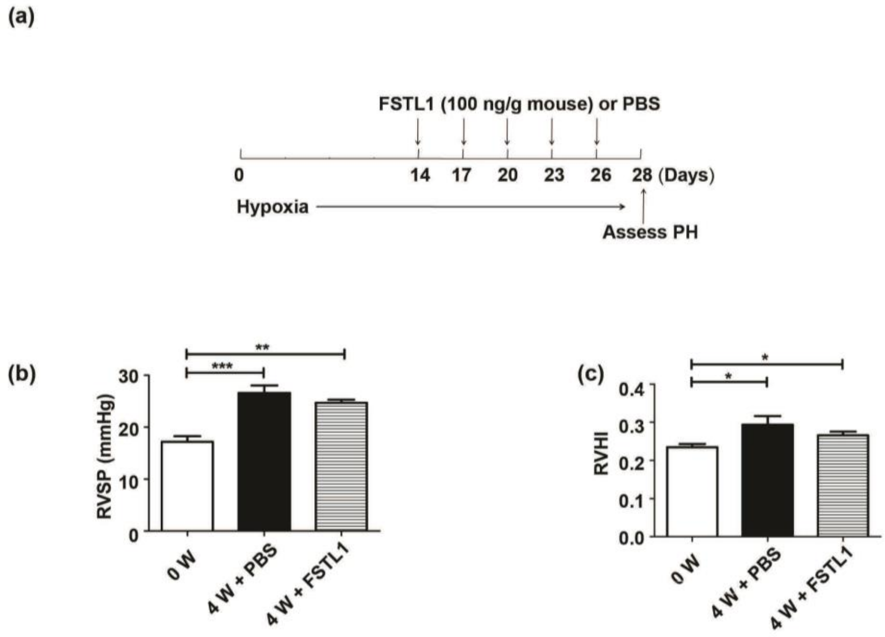

Supplementary Figure S2. No significantly attenuated HPH is observed by administration of FSTL1 after 2 weeks of hypoxia in mice. (a) Intervention regimen of FSTL1 in HPH model of mice. (b) RVSP and RVHI (c) in mice intravenously administrated with FSTL1 or PBS after 2 weeks of hypoxia. n = 5. Data are presented as mean ± SEM. * $P < 0.05$, ** $P < 0.01$, *** $P < 0.001$. HPH = hypoxia-induced PH. PBS = phosphate buffer saline. RVSP = right ventricular systolic pressure. RVHI = right ventricular hypertrophy index. W = week.

Original western blots of figures

Figure 1e

FSTL1

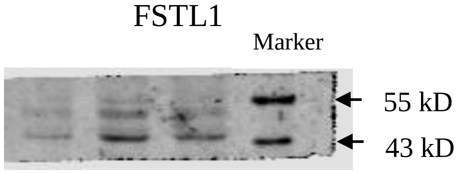

GAPDH

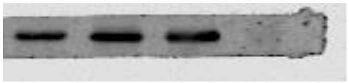

Figure 2a

FSTL1 (serum)

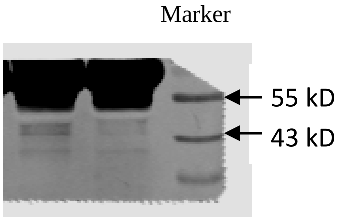

FSTL1

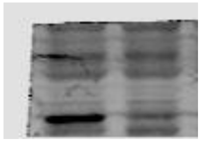

GAPDH

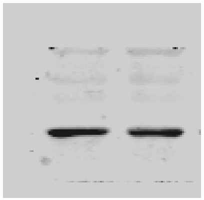

Figure 3b

FSTL1

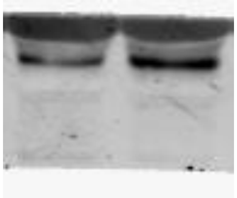

Figure 5b

FSTL1 (media)

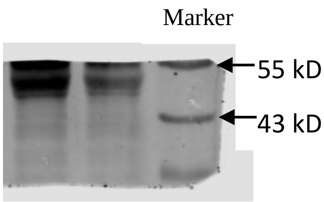

FSTL1

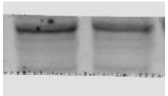

GAPDH

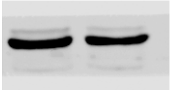

Figure 6a

P-AMPK

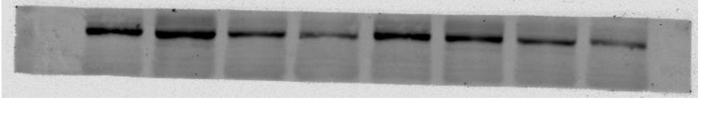

T-AMPK

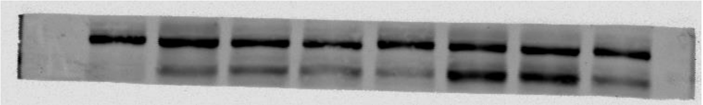

P-ERK

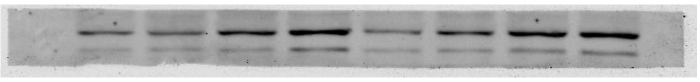

T-ERK

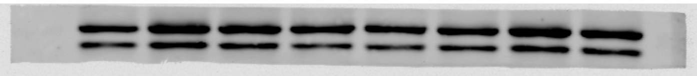

GAPDH

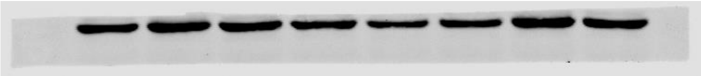

Figure 6b

P-AMPK

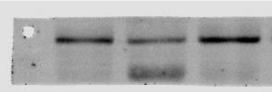

T-AMPK

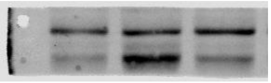

P-ERK

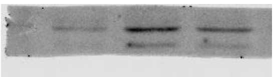

T-ERK

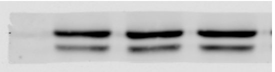

GAPDH

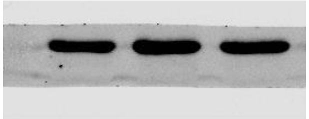

Figure 6c

P-ERK

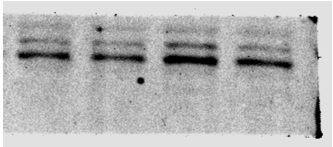

T-ERK

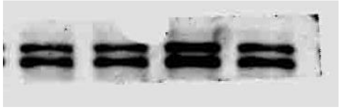

GAPDH

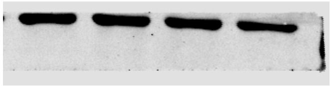

Figure 6d

P-AMPK

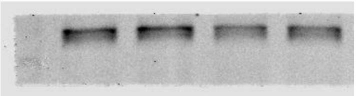

T-AMPK

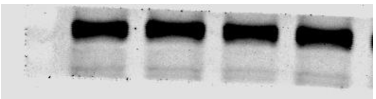

GAPDH

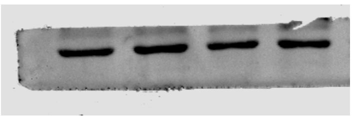

Figure 6e

P-ERK

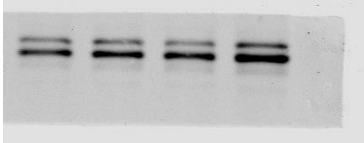

T-ERK

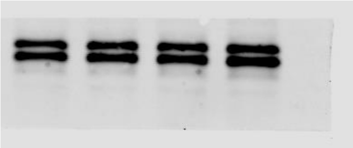

GAPDH

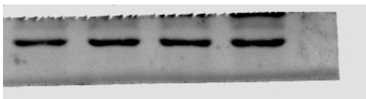

Supplementary Figure S1a

P-Smad 1/5/8

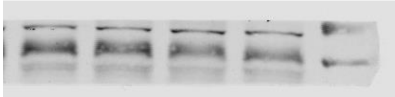

T-Smad 1/5/8

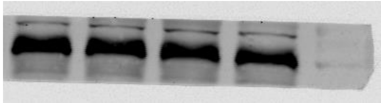

GAPDH

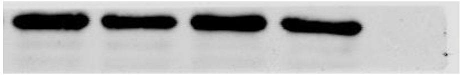

P-p38

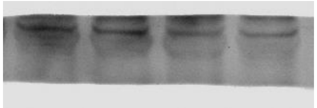

T-p38

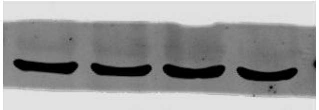

GAPDH

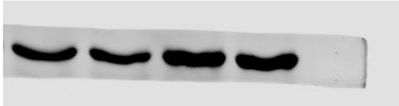

P-JNK

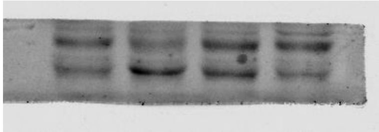

T-JNK

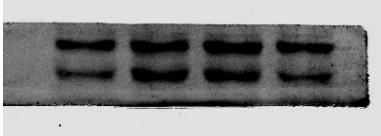

GAPDH

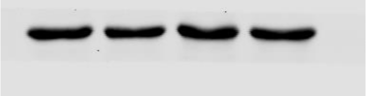

Supplementary Figure S1b

P-Smad 1/5/8

HPASMCs HPAECs

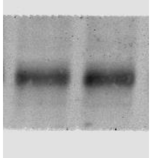

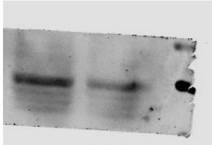

T-Smad 1/5/8

HPASMCs HPAECs

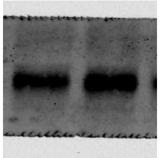

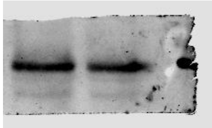

GAPDH

HPASMCs

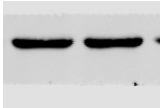

HPAECs

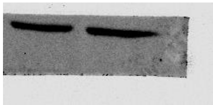

Supplement: Supplementary Tables and Figures [file srep45820-s1.pdf]
